# Catastrophic Household Expenditure Associated with Out-of-Pocket Healthcare Payments in Spain

**DOI:** 10.3390/ijerph18030932

**Published:** 2021-01-21

**Authors:** Samuel López-López, Raúl del Pozo-Rubio, Marta Ortega-Ortega, Francisco Escribano-Sotos

**Affiliations:** 1 Castilla-La Mancha Health Services, SESCAM, Hospital of Cuenca, C/Hermandad de Donantes de Sangre, 1, 16002 Cuenca, Spain; 2 Department of Economic Analysis and Finance, University of Castilla-La Mancha, Avda. Los Alfares, 44, 16071 Cuenca, Spain; raul.delpozo@uclm.es; 3 Research Group on Food, Economy and Society, University of Castilla-La Mancha, Avda. Los Alfares, 44, 16071 Cuenca, Spain; francisco.esotos@uclm.es; 4 Department of Applied and Public Economics, and Political Economy, Complutense University of Madrid, Campus de Somosaguas s/n, Pozuelo de Alarcón, 28223 Madrid, Spain; martao@ucm.es; 5 Department of Economic Analysis and Finance, University of Castilla-La Mancha, Plaza de la Universidad s/n, 02001 Albacete, Spain

**Keywords:** Spain, out-of-pocket payments, health, catastrophic, health insurance, households, economic crisis

## Abstract

Background. The financial effect of households’ out-of-pocket payments (OOP) on access and use of health systems has been extensively studied in the literature, especially in emerging or developing countries. However, it has been the subject of little research in European countries, and is almost nonexistent after the financial crisis of 2008. The aim of the work is to analyze the incidence and intensity of financial catastrophism derived from Spanish households’ out-of-pocket payments associated with health care during the period 2008–2015. Methods. The Household Budget Survey was used and catastrophic measures were estimated, classifying the households into those above the threshold of catastrophe versus below. Three ordered logistic regression models and margins effects were estimated. Results. The results reveal that, in 2008, 4.42% of Spanish households dedicated more than 40% of their income to financing out-of-pocket payments in health, with an average annual gap of EUR 259.84 (DE: EUR 2431.55), which in overall terms amounts to EUR 3939.44 million (0.36% of GDP). Conclusion. The findings of this study reveal the existence of catastrophic households resulting from OOP payments associated with health care in Spain and the need to design financial protection policies against the financial risk derived from facing these types of costs.

## 1. Introduction 

Health protection is one of the rights included in the 1948 Universal Declaration of Human Rights, whereby everyone has the right to health, medical care, and security in the event of sickness [1]. In fact, Universal Health Coverage is one of the 13 strategic aims within the Sustainable Development Goals set by the World Health Organization for 2030 [2].

However, the population’s access to healthcare systems in most countries requires families to make monetary payments, be it in the form of fees, copayment, taxes, or other types of contributions [2]: these payments are known as out-of-pocket payments (OPPs) [3]. Such OPPs can cause significant financial problems for families [4,5], especially those resident in low- or middle-income countries [6], in communities or regions with lower per capita income [7,8], in low-income families [9], in countries with an aging population [10], or in families with unemployed members [11]. Although copayment can help curb unnecessary access to health services and raise people’s awareness of misuse of health care, it can also lead to a lack of financial resources making it impossible for some individuals to access the system when needed [12,13].

The onset of the economic crisis marked by the collapse of Lehman Brothers in 2008 triggered the need for governments across the world to adopt large-scale legislation with substantive financial measures to tackle the situation [14]. Europe was not spared the effects of the crisis, nor was Spain, alongside countries such as Italy, Greece, Portugal, and Ireland [15]. The effects of the recession were especially severe in Spain due to two internal factors, namely excessive private sector borrowing (largely externally funded) and a significant concentration of financial resources in the construction industry [16]. The combination of both factors, together with the tense international scenario, led to high levels of public deficit and debt [16].

The economic crisis in Spain can be divided into three phases: the first phase marked by a significant drop in economic activity between the third quarter of 2008 and 2009; a second phase of stagnation between 2010 and the first quarter of 2011, and a third phase of steep recession and fall in gross domestic product (GDP) from 2011 to 2013 [16]. Spain’s fiscal legislative response followed a similar pattern, reacting to each of the phases: 2008–2009, by adopting counter-cyclical measures and others to increase government spending; 2010–2011: a first phase of measures of fiscal adjustment and consolidation; from 2012, by adopting strict structural fiscal measures [16], in which the Spanish healthcare sector was particularly mistreated in order to reduce the budget deficit and meet the objectives of fiscal consolidation [17,18]. Specifically, the most prominent measures corresponded to three blocks: changes to taxes and services, modifications to items of government spending, and structural fiscal reform [19]. 

Among the modification to public spending, the measures implemented with regard to health care reduced its weight in GDP by 0.6% in the period 2010–2013, and by 0.8% in the period 2013–2018, while this had previously increased by 0.9% of GDP in the period 2007–2010 [19]. It is important to note that the redistributive effect of education and health public spending is estimated to entail a reduction of 20% in the inequality of income distribution in Spain [18]. The healthcare reforms undertaken during the period 2008–2013 can be divided into three groups: policies intended to change the level of contributions to publicly financed health care, policies intended to affect the volume and quality of publicly financed health care, and policies intended to affect costs [20]. Among the healthcare reforms, we can highlight changes to right holders, both those insured and beneficiaries, to the classification of services and prescribed medications and pharmaceutical products, and modifications to the contribution of beneficiaries to the cost of prescriptions [21,22,23,24,25]. The only cost sharing in the Spanish public health system is that related to the cost of medications. This cost was increased by 10% of the cost of the medication for those who are working and their annual income exceeds EUR 18,000, and by 20% for those whose annual income exceeds EUR 100,000. In the case of pensioners, the increase is also 10%, with a limit of 8 and 18 euros per month for those whose income is less than EUR 18,000 and more than EUR 18,000, respectively. In the case of pensioners with incomes over EUR 100,000, the cost sharing is 60% with a monthly limit of EUR 60. The cost sharing of long-term unemployed and noncontributory pensioners’ remains null [20]. Other measures were outsourcing of services (increase in agreements with the private sector), reduction of workforce (retirements without replacement or low percentage of replacement of vacancies), reduction and concentration of sanitary areas, closure of services (rural emergencies), and concentration of services (laboratories) [18]. The immediate effect of the set of measures was the reduction of the health budgets of both the central and regional governments, with a reduction of 13.7% and 22.6%, respectively [20]. In Europe, it has been reported that during the recession, every USD 100 less obtained in tax revenue led to a USD 2.72 reduction in public health expenditure [26].

The impact of the financial crisis in Spain generated an enormous increase in unemployment, which rose from 8% in the second quarter of 2008 to 26% in the fourth quarter of 2012 [27]. GDP fell by 9% over the period 2008–2013 [15], and inequality in terms of income was acute; in the period 2008–2013, 34% of the individuals in the lowest-income decile witnessed a reduction in their earnings in real terms, while in the highest-income decile, the proportion whose income fell was 16% [15]. Despite all the measures implemented, the public deficit in 2008 was −5.4%, reaching a maximum of −9.5% in 2009. It was still −4.5% in 2013 [28].

The aim of the present study was to analyze the percentage of households and intensity of catastrophism derived from OPPs associated with health care in Spain during the period 2008–2015, together with the sociodemographic and economic factors associated with this catastrophism. To the best of our knowledge, no studies have analyzed the catastrophic effect of OPPs for health in Spain, either now or during the economic crisis, arguably because Spain has a robust healthcare system characterized by a National Health System model based on public provision of health services, being among the leading 20 systems in the world [29].

### 1.1. Catastrophic Expenditure on Out-of-Pocket Healthcare Payments 

#### Review of the Literature

One of the most widely used tools to capture financial tensions in households generated by copayments for access or use of healthcare systems is so-called financial catastrophism [7,9]. A catastrophic household is defined as one where the total amount of financial resources the household is obliged to dedicate to health care exceeds a certain percentage (which we shall call threshold) of the equivalent household income [30]. These thresholds are normative, and may vary depending on the health system or sickness, country or moment of time. The most commonly used thresholds are 10%, 20%, 30%, and 40% [7,10,31]. Two recent studies, focused on long-term care of older adults with chronic diseases in China [10] and persons with cancer in Spain [32] have introduced higher thresholds than those traditionally used, 60% and 100%, respectively.

The catastrophic effect of OPPs for health care has been the subject of extensive study, especially in countries in Asia, such as Vietnam [7], Nepal [33], Iran [34], Thailand [35], Turkey [36], Bangladesh [37], South Korea [38], and China [39], including systematic reviews of the Asian continent [40,41]. A number of studies have also been conducted in African countries, such as Nigeria [42], Zambia [43], and Kenya [44], as well as systematic reviews [45]. 

Studies have also been carried out in countries in Latin America [46,47]. A recent systematic review showed that the number of such works in Europe is limited and the data obsolete [48]. The European countries where analyses have been carried out in overall terms of OPPs for health are Portugal [49], Poland, Germany and Denmark [50], Italy [51], and in the private healthcare sector in Greece [52].

There is an especially extensive body of literature on specific areas of health care, including analyses of specific population groups, such as older persons [53], older persons with chronic diseases [10,54], persons with disabilities [55,56], as well as works on the acquisition of medication [57] or diseases such as cancer [4,32] or HIV [58]. 

The findings of all these studies are as diverse as the intrinsic circumstances and characteristics of each country, population group, and case study under analysis. In general terms, a recent systematic review conducted in 133 countries underlined that, in 2010, the global incidence rate of catastrophic expenditure was 11.7%, revealing that 808 million people had incurred catastrophic health expenses [59].

In Spain, this methodology has only been used to analyze the catastrophic effect of OPPs for long-term care, reporting that 68.07% of families are obliged to devote more than 40% of their income to such OPPs, with a mean monthly gap of EUR 341.66 for level III, the most severe level of dependence [60]. No study, however, has been conducted on healthcare expenditure.

## 2. Materials and Methodology

### 2.1. Data

The data used were taken from the Household Budget Survey (HBS) [61]. This survey consists of an annual questionnaire administered to more than 20,000 households (a total of 175,943 households between 2008 and 2015), which collects socioeconomic information on standard of living, income, and the head of the household’s job, although its added value lies in the details of the households’ annual consumer spending (equipment, housing, food, health care, education, tourism). The classification used to capture expenses is the COICOP (Classification of Individual Consumption by Purpose), comprising the following categories: 1. Food and nonalcoholic beverages; 2. Alcoholic beverages, tobacco and narcotics; 3. Clothing and footwear; 4. Housing, water, electricity, gas, and other fuels; 5. Furniture. Household equipment and ordinary expenses for the maintenance of the dwelling; 6. Health; 7. Transport; 8. Communication; 9. Leisure, performances and culture; 10. Education; 11. Restaurants, cafés and hotels; 12. Miscellaneous goods and services.

Of the above categories, the sixth refers to the overall health expenditure families must make. The specific subgroups of this category are as follows: therapeutic appliances and equipment, medical services, dental services, services of medical analysis and X-rays, outpatient medical services, other outpatient services, hospital services, and nonspecific health expenses. This information is collected on a quarterly basis, and then categorized by codes and annualized by the Spanish National Statistics Institute for its final presentation. The overall amount spent on Category 6 forms the OPPs analyzed in the present study and the amount of expenditure made by households for reasons of health.

In addition, we use the households’ declared incomes, which were converted into equivalized household incomes. To this end, and following previous studies [7,11], we calculated the equivalized consumer units, or equivalized household members, using the modified OECD scale, which assigns a value of 1 to the first household member, 0.5 to each household member aged over 13 years (14 or more), and 0.3 to each member aged 13 or under [62]. Subsequently, the income per consumer unit or equivalized member is obtained by dividing the total household income by the number of consumer units or equivalized household members.

Finally, we obtained the sociodemographic characteristics of the main breadwinner in each household, detailed in the following section.

### 2.2. Catastrophism Measure

Following the methodology proposed by Wagstaff and van Doorslaer (2003) [7], we defined a dummy variable E_i_, which takes the value of 1 when the OPPs_i_ of household i, as a proportion of household income i (y_i_) exceeds the normative threshold (z_cat_), that is, (OPPs_i_/y_i_) > z_cat_, and 0 otherwise. The household is thus defined as catastrophic when its OPPs for health care exceed the threshold. The number of households in a catastrophic situation or *rate of catastrophic households due to healthcare OPPs* (Hcat), was defined as:

(1) $$H_{cat}=\frac{1}{N}\overset{n}{\underset{i=1}{\sum }}E_{i}={\bar{x}}_{E}$$

where *n* is the sample size, and ${\bar{x}}_{E}$ is the mean sample of E_i_. In addition, we defined the *mean catastrophic gap of households due to health payments* (O_i_) as the difference between the OPPs made by the household and the normative threshold used (z_cat_) multiplied by the equivalized household income, that is, OPPs − z_cat_ * y_i_ si E_i_ = 1, or 0 otherwise (i.e., the catastrophic gap is the difference between the value of total OOP financed by the household and the normative threshold used corresponding to each household valued in euros). This measure represents the intensity of the catastrophic gap due to OOPs on health care, defined as:

(2) $$O_{cat}=\frac{1}{n}\overset{n}{\underset{i=1}{\sum }}O_{i}={\bar{x}}_{AO}$$

Consequently, the total catastrophic gap due to OPPs for health care (GO_i_) was defined as:

(3) $$GO_{cat}=\overset{n}{\underset{i=1}{\sum }}O_{i}$$

Therefore, while the incidence variable (H_cat_) reflects the proportion of households that exceed the normative threshold determined as a result of OPPs for health care, the intensity variable (O_cat_) indicates the mean overall amount in absolute values that exceeds the normative threshold established. The present work implemented the normative thresholds most widely used in the literature (z_cat_), namely 10%, 20%, 30%, and 40%.

#### Factors Associated with Catastrophism

Once we had classified households in terms of their OPPs having exceeded the corresponding normative threshold, and following the study aims, we estimated an ordered logistic regression model (one for each year), due the ordinal nature of the dependent variable. To this end, we used the *rate of catastrophic households due to healthcare OPPs* (Hcat) as dependent variable (y_i_ = 1, if the OPPs for health care do not exceed 10% of household equivalized income; y_i_ = 2, if they exceed 10% of household equivalized income but not 20%; y_i_ = 3, if they exceed 20% of household equivalized income but not 30%; y_i_ = 4, if they exceed 30% of household equivalized income but not 40%; y_i_ = 5, if they exceed 40% of household equivalized income, with i = 1, …, *n*. where *n* is each of the households comprising the sample). The models were estimated following the methodology proposed by Williams (2006) [63] using the Stata command gologit2 with other specifications (autofit, lrforce, pl and npl, etc.). This methodology allows three special cases of the generalized model to be fitted: the proportional odds/parallel-lines model, the partial proportional odds model, and the logistic regression model. It also detects collinear predictors and drops them automatically.

Estimating this model allowed us to determine the sociodemographic factors whose parameters were statistically significant and that were associated with situations of catastrophic expenditure due to OPPS for health care. To reflect this association, the marginal effects on the set of variables included in the analysis were estimated.

In the set of explanatory variables, we included those recommended in the literature and the categories established in each one [40,54,64,65]. The sociodemographic variables incorporated into the model referred to the head of the household, being (the reference category is indicated by *): gender (man*; woman); age (below 65 years*; between 65 and 74 years; between 75 and 84 years; over 85 years); marital status (married*; single; widowed, separated/divorced); educational level (low educational level* (primary education unfinished, finished or equivalent); medium educational level (compulsory secondary education/higher secondary education/intermediate level vocational training); high educational level (university education or equivalent)); employment situation (employed*; unemployed; pensioner or retired; other situations (homemaker, students, others)); monthly household income (less than EUR 1200*; between EUR 1200 and 2500; more than EUR 2500); per capita GDP in the autonomous community of residence (low per capita GDP* (Extremadura, Andalusia, Castilla-La Mancha, Region of Murcia, Canary Islands, Ceuta and Melilla); medium per capita GDP (Valencian Community, Galicia, Asturias, Castilla-León, Cantabria, Balearic Islands); high per capita GDP (La Rioja, Aragon, Catalonia, Navarre, Madrid and the Basque Country)); finally, place of residence (rural*; urban). The coding of the variables is included in Table S1 (see Supplementary Material)

As the results obtained are intrinsically similar across the different waves of the survey, the present study only includes the descriptive statistics and the results obtained for the marginal effects for 2008, 2011, and 2015, which capture the start, middle, and end of the economic crisis.

All the calculations were conducted using the statistical software Stata 13.0 (StataCorp LP, College Station, TX, USA).

## 3. Results

Table 1 shows the sociodemographic information for 2008. Our findings show that 72.66% of households had a man as the head, with the age being predominantly under 65 years (75.36%), and the majority marital status being married (60.62%). Smaller differences were found in the category of educational level, with the medium level being predominant (43.97%), followed by low (30.21%), and finally, high level of education (25.82%). Almost two thirds of the respondents were employed (62.28%), followed by the economic status of pensioner or retired (28.04%). A total of 41.24% of the households had a monthly income below EUR 1200, while a similar proportion (42.42%) reported an income in the EUR 1200–2500 range. Three of every 10 people in Spain were found to be living in an autonomous community with a low per capita GDP (31.02%), while 29.02% lived in a medium per capita GDP community. Of the sample, 84.67% lived in an urban area.

Regarding the disaggregation of variables according to the catastrophism thresholds, the sample is highly similar in the case of thresholds below 40%, while for the 10% threshold, changes were observed in the distribution of the sample according to certain variables. For example, the number of households with a male head is slightly lower (a 4.42% decrease), while the age of the head of the household increases in all the ranges above 65 years, as does the percentage of heads of households who are separated or divorced (3.22% increase) and widowed (4.13% increase). Especially striking is the increase in households with a low educational level (7.46%) compared to the fall in those with a high level (−6.90%). Finally, there was a considerable increase in the pensioner or retired employment category (13.30%), but, above all, there was a notable increase of 22.20% in households whose monthly income was below EUR 1200, while those whose income was between EUR 1200 and 1500 decreased by 10.94%.

Table 2 and Table 3 show the evolution of the sample’s sociodemographic characteristics for 2011 and 2015. The pattern is similar to that of 2008, but with the following differences. There is a decrease in the number of households whose head is a man, together with a slight reduction in the age of the breadwinner in the successive waves. There is also a decline in the percentage of households with a lower educational level and a corresponding rise in those with medium and high levels. It is worth noting the increase in 2011 in the proportion of married heads of households devoting 40% of the household income to OPPs for health care.

Table 4 shows the effects of catastrophic out-of-pocket payments on health in Spanish households, in terms of both incidence and intensity. It can be seen that, in 2008, 21.32% of households devoted more than 10% of their monthly income to healthcare OPPs, with a mean annual catastrophic gap of EUR 582.04 (SD: EUR 2967.03). Both measures decrease as the normative thresholds increase. Thus, 4.42% of households in Spain spent more than 40% of their income to fund OPPs for health, with a mean annual catastrophic gap of EUR 259.84 (SD: EUR 2431.55). Overall, at national level, the annual catastrophic gap resulting from devoting more than 10% of household income to pay healthcare OPPs is EUR 9075.14 million (0.82% GDP). This amount falls to EUR 6357.78 million (0.57% GDP), EUR 4881.70 million (0.44% GDP), and EUR 3939.44 million (0.36% GDP) for the thresholds of 20%, 30%, and 40%, respectively. Finally, the evolution of the same measures over the years from 2009 to 2015 reveals amounts that remain practically constant over the period under analysis.

Table 5 shows the marginal effects derived from the ordered logistic regression for 2008, with all effects revealing statistically significant parameters. In general terms, in all the thresholds above 10%, it can be seen that the results are intrinsically similar but different to those of the below 10% threshold. Among the factors associated with financial catastrophism due to OPPs for health care in the 40% threshold are being female (0.19%), being aged between 65 and 74 (0.59%), 75 and 84 (0.69%), and over 85 years (1.79%), being married, medium level (0.23%) and high level of education (0.19%), any other employment status than employed, having a monthly income of less than EUR 1200, living in an urban area, and living in an autonomous community with medium or high per capita GDP.

Table 6 and Table 7 present the analyses for the 2011 and 2015 waves, respectively. For both years, the amounts and patterns of behavior are very similar to those for 2008.

The classification accuracy of the models estimated is 88.99%, 80.45%, and 88.52% for the years 2008, 2011, and 2015, respectively.

## 4. Discussion

Although the Spanish National Health System was recognized as one of the most robust in the world in 2015, being placed 12th in the world ranking of health systems [66], the results of the present study reveal a limited financial scope and the existence of a lower, but not inconsiderable, percentage of households that suffer catastrophic effects of being obliged to make OPPs for health care.

Xu et al. (2003) [9], in their study of 59 countries worldwide, found that the percentage of households facing catastrophic healthcare OPPs for health care varied from 0.01% in the Czech Republic to a maximum of 10.45% in Vietnam. They reported that countries with advanced structures of social insurance or tax-funded health systems protect their population from catastrophic payments, and among such countries the incidence rate of households facing catastrophic spending was 0.5% or more in countries such as Portugal, Greece, Switzerland, and the USA. Spain, with an incidence of 0.48% can be included in this group. The underestimation of the rate in Spain was due to the definition of catastrophic payments used compared with that proposed in the present study, where other categories have been included, such as dental services, outpatient medical services, other outpatient services, and nonspecific health expenses. Subsequently, Xu et al. (2007) [64], in their review of 89 countries that account for 89% of the world’s population, reported a global incidence rate of 1.47%, with only 18 countries with an incidence of above 4%.

The incidence rate found for Spain with a mean of around 4% in the 40% threshold for all the years analyzed is similar to the results obtained for Portugal, where the rate was 5.03% in 2005, with significant differences between regions [49], and lower than Poland, which had an incidence of 8.8% for the period 2000–2010; these figures are far removed from the rates of 0.4% in Germany and 0.8% in Denmark for the same period under analysis [50]. It is worth noting that the results for Greece are significantly higher, with an incidence rate of 9.75% for the 25% threshold (highest threshold analyzed), although, in this case, the analysis focused on insurance and payments for access to the private healthcare market [52].

Among the sociodemographic characteristics, we found that being female, being over 65 years of age, and being married are factors that increase the likelihood of incurring catastrophic healthcare expenditure, which is consistent with previous studies [54,64,67,68]. While the gender factor varies according to the country analyzed [61,62], countries with a larger number of persons in the older age groups have an increased likelihood of generating catastrophic financial payments [49,52,64]. It is worth noting that the marital status variable has rarely been examined in the literature of this kind, although this variable was included in a study on households where one of the members has disabilities, reporting similar results to those of the present work [69]. 

Households with lower income levels and any other employment status than employed have a greater likelihood of incurring catastrophic payments [70], while the likelihood of suffering financial catastrophism is multiplied by four or five [55] and as much as by 16 [67] in the case of individuals in the lowest income percentiles. The likelihood of catastrophic expenditure in individuals assessed as incapable of work or who have retired due to disability increases by 1.41 and 1.14 times, respectively [49], and in the case of homemakers, this likelihood is multiplied 1.51 times [67]. 

One of the most striking findings is that higher levels of education are associated with a greater likelihood of catastrophic expenditure. Although some studies report no conclusive results [54,68,70], a study on persons with disabilities in South Korea showed that people with higher educational levels were more likely to suffer financial catastrophism. This likelihood was specifically 3.6 and 1.6 times higher for medium and higher levels of education, respectively, taking primary education as the reference [67].

Finally, living in an urban area (compared to a rural environment) increases the likelihood of catastrophic expenditure, which is in line with countries such as Portugal [49] or China [10]. The same is true in the case of living in autonomous communities in Spain with medium or high per capita GDP [54,64].

In summary, the sociodemographic profile associated with financial catastrophism in Spain is similar to that described for countries such as Denmark, Germany, or Poland, where the population groups most prone to incurring catastrophic expenditure are individuals with fewer resources, women, and older adults [53]. It is worth highlighting that a recent systematic review concluded that when formal fees are introduced for families’ access to health care, financial protection against catastrophic OPPs for medical care is required for the most vulnerable groups in the population [12].

## 5. Conclusions

To the best of our knowledge, this is the first study to analyze the sociodemographic factors (magnitude and sign) associated with suffering a situation of financial catastrophism as a result of OPPs for health care in Spain, and to assess the historical development of these during the period of the last economic crisis.

The findings reveal that being female, being in an older age group, being married, having a medium or high level of education, and living in an urban area or in an autonomous community with higher GDP increase the likelihood of catastrophic OPPs for health in Spanish households. Our findings suggest that legislators should design health policies that include the aim of financially protecting such catastrophic households, establishing measures for specific profiles and/or certain income levels that keep these households safe from situations of catastrophism. 

The analysis of the historical data reveals a robust consistency in the measures analyzed, despite the large number of reforms implemented in the areas of the economy and health. It is striking that although the fiscal cutbacks adopted in Spain appear to have generated bottlenecks in the provision of certain public goods and services, such as those related to health care, with important social programs having been suspended [19], this is not reflected in our findings. It is necessary, however, to consider significant financial protection for individuals over the age of 50 in European health systems following the great recession [71], due to the weakening of such systems at the onset of the crisis, and taking into account that the level of financial protection varies across European countries [48]. Therefore, any consideration of types of copayment should take into account minimum income levels for exemption from charges.

The primary limitation of this work is that we were unable to consider the different waves of the survey as panel data, because the households in the survey change every 24 months. This limited the econometric analysis of the data, which had to be individually processed year by year, and thus it was impossible to follow the evolution of the households over the period of analysis. Nonetheless, this is the first study within the Spanish health system to analyze the financial impact of OPPs made by families for health care.

Future research is needed that delves deeper into the health expenditure profiles of households in Spain, given that studies with this type of analysis in Spain and Europe are scant, and just a minority have examined the likelihood of incurring financial difficulties and what drives the lack of financial protection [48]. In this sense, it is necessary to analyze firstly, the specific components of OPPs for health to determine those with a greater impact in terms of financial catastrophism; secondly, to detect what threshold (normative or not) causes households to start having difficulties in securing basic life needs; thirdly, setting different welfare levels and determining how the OOPs affect them. All of this is needed in order to subsequently be able to formulate health policy decisions aimed at the financial coverage of the profiles described in the present study. 

## Figures and Tables

**Table 1:** Descriptive statistics of the sociodemographic variables of households in Spain based on the percentage of resources they dedicate to health payments. Year 2008.

	Total	Threshold < 10%	10–20%	20–30%	30–40%	Threshold > 40%
Gender						
Male	72.66%	73.05%	71.04%	65.84%	68.53%	68.24%
Female	27.34%	26.95%	28.96%	34.16%	31.47%	31.76%
Head’s household age						
Less than 65	75.36%	76.13%	72.70%	65.67%	64.16%	62.36%
65–74	12.57%	12.01%	15.19%	20.62%	22.77%	17.57%
75–84	10.76%	10.62%	10.69%	12.25%	11.30%	16.04%
More than 85	1.31%	1.24%	1.42%	1.46%	1.77%	4.03%
Marital status						
Married	60.62%	60.40%	64.51%	63.11%	59.06%	56.49%
Single	19.61%	20.17%	14.94%	14.35%	13.97%	16.39%
Separated/Divorced	12.74%	12.61%	12.45%	14.08%	17.63%	15.96%
Widowed	7.03%	6.82%	8.10%	8.46%	9.34%	11.16%
Educational level						
Low level (primary school incomplete, primary or equivalent)	30.21%	29.81%	32.91%	34.60%	27.95%	37.67%
Middle level (secondary school/middle level professional)	43.97%	43.87%	45.03%	43.65%	47.95%	43.41%
University degree or equivalent (university degree or equivalent)	25.82%	26.32%	22.06%	21.75%	24.10%	18.92%
Activity status						
Employed	62.28%	63.28%	58.92%	45.83%	48.15%	48.64%
Unemployed	4.73%	4.66%	4.80%	8.01%	3.66%	5.18%
Receiving earnings-related pension	28.04%	27.24%	30.49%	39.28%	40.23%	41.34%
Other situations (homecare, student, etc.)	4.95%	4.82%	5.79%	6.88%	7.96%	4.84%
Household monthly income						
Low level income (less than EUR 1200)	41.24%	39.73%	51.00%	54.71%	53.64%	63.44%
Middle level income (EUR 1200–2500)	42.42%	42.96%	40.84%	34.26%	39.12%	31.48%
High level income (more than EUR 2500)	16.34%	17.31%	8.16%	11.03%	7.24%	5.08%
GDP per capita						
Low GDP	31.02%	31.00%	32.58%	28.94%	31.45%	29.04%
Middle GDP per capita	29.02%	28.91%	29.94%	28.06%	26.10%	34.05%
High GDP per capita	39.96%	40.09%	37.48%	43.00%	42.45%	36.91%
Place of residence						
Rural	15.33%	15.34%	15.68%	15.40%	15.55%	13.25%
Urban	84.67%	84.66%	84.32%	84.60%	84.45%	86.75%
*n*	22.021	19.596	1.380	439	204	402

**Table 2:** Descriptive statistics of the sociodemographic variables of households in Spain based on the percentage of resources they dedicate to health payments. Year 2011.

	Total	Threshold < 10%	10% ≤ Threshold < 20%	20% ≤ Threshold < 30%	30% ≤ Threshold < 40%	Threshold ≥ 40%
Gender						
Male	69.28%	68.62%	71.31%	72.48%	70.25%	75.43%
Female	30.72%	28.69%	62.18%	27.52%	29.75%	24.57%
Head’s household age						
Less than 65	76.17%	75.94%	81.09%	74.88%	72.92%	70.91%
65–74	12.37%	12.28%	11.05%	12.62%	15.16%	16.67%
75–84	8.88%	9.05%	6.15%	10.74%	10.92%	9.36%
More than 85	2.58%	2.73%	1.71%	1.76%	1.00%	3.06%
Marital status						
Married	57.32%	54.60%	69.55%	66.30%	70.88%	72.44%
Single	21.21%	23.05%	13.37%	14.85%	10.84%	10.73%
Separated/Divorced	12.50%	13.19%	8.33%	11.44%	9.19%	10.26%
Widowed	8.97%	9.16%	8.75%	7.41%	9.09%	6.57%
Educational level						
Low level (primary school incomplete, primary or equivalent)	20.99%	21.18%	16.45%	24.36%	21.32%	25.02%
Middle level (secondary school/middle level professional)	50.16%	49.54%	53.82%	51.22%	52.68%	52.55%
University degree or equivalent (university degree or equivalent)	28.85%	29.28%	29.73%	24.42%	26.00%	22.43%
Activity status						
Employed	57.58%	57.68%	62.41%	54.21%	53.67%	47.58%
Unemployed	8.86%	8.65%	9.77%	8.70%	12.18%	9.78%
Receiving earnings-related pension	27.74%	27.52%	24.02%	31.35%	30.75%	37.92%
Other situations (homecare, student, etc.)	5.82%	6.15%	3.80%	5.74%	3.40%	4.72%
Household monthly income						
Low level income (less than EUR 1200)	44.67%	44.60%	40.93%	50.32%	47.70%	48.86%
Middle level income (EUR 1200–2500)	40.24%	39.87%	43.77%	39.13%	40.88%	40.55%
High level income (more than EUR 2500)	15.09%	15.53%	15.30%	10.55%	11.42%	10.59%
GDP per capita						
Low GDP	31.26%	30.88%	32.47%	35.17%	34.12%	31.25%
Middle GDP per capita	28.79%	28.99%	28.03%	27.34%	32.60%	25.84%
High GDP per capita	39.95%	40.13%	39.50%	37.49%	33.28%	42.91%
Place of residence						
Rural	14.91%	14.82%	13.56%	16.47%	18.59%	17.24%
Urban	85.09%	85.18%	86.44%	83.53%	81.41%	82.76%
*n*	21,625	17,399	2143	857	407	819

**Table 3:** Descriptive statistics of the sociodemographic variables of households in Spain based on the percentage of resources they dedicate to health payments. Year 2015.

	Total	Threshold < 10%	10% ≤ Threshold < 20%	20% ≤ Threshold < 30%	30% ≤ Threshold < 40%	Threshold ≥ 40%
Gender						
Male	66.25%	66.41%	63.31%	69.03%	67.41%	64.55%
Female	33.75%	33.59%	36.69%	30.97%	32.59%	35.45%
Head’s household age						
Less than 65	72.63%	73.76%	65.23%	69.08%	64.29%	52.62%
65–74	13.62%	13.14%	15.78%	16.13%	17.96%	24.28%
75–84	9.34%	8.96%	12.65%	9.48%	11.96%	14.92%
More than 85	3.16%	3.00%	4.51%	4.27%	1.39%	5.79%
Marital status						
Married	54.02%	53.86%	55.60%	56.51%	53.62%	53.06%
Single	22.85%	23.45%	20.37%	16.87%	13.37%	14.57%
Separated/Divorced	12.92%	12.63%	14.64%	11.12%	22.08%	18.64%
Widowed	10.21%	10.06%	9.39%	15.50%	10.93%	13.73%
Educational level						
Low level (primary school incomplete, primary or equivalent)	19.80%	19.28%	23.19%	22.13%	24.26%	28.33%
Middle level (secondary school/middle level professional)	48.82%	48.63%	49.52%	47.74%	53.65%	54.31%
University degree or equivalent (university degree or equivalent)	31.38%	32.09%	27.29%	30.13%	22.09%	17.36%
Activity status						
Employed	56.78%	58.23%	49.91%	45.32%	41.33%	32.48%
Unemployed	8.24%	8.29%	6.18%	12.72%	5.56%	8.46%
Receiving earnings-related pension	29.22%	27.97%	35.25%	37.90%	44.95%	50.19%
Other situations (homecare, student, etc.)	5.76%	5.51%	8.66%	4.06%	8.16%	8.87%
Household monthly income						
Low level income (less than EUR 1200)	46.54%	45.27%	53.82%	57.08%	58.13%	64.03%
Middle level income (EUR 1200–2500)	39.98%	40.46%	37.92%	36.14%	34.26%	31.23%
High level income (more than EUR 2500)	13.48%	14.27%	8.26%	6.78%	7.61%	4.74%
GDP per capita						
Low GDP	31.54%	31.46%	32.03%	34.46%	36.80%	27.76%
Middle GDP per capita	28.60%	28.55%	28.00%	29.47%	31.24%	30.82%
High GDP per capita	39.86%	39.99%	39.97%	36.07%	31.96%	41.42%
Place of residence						
Rural	14.37%	14.36%	13.57%	14.87%	16.88%	15.82%
Urban	85.63%	85.64%	86.43%	85.13%	83.12%	84.18%
*n*	22,054	19,524	1385	498	212	435

**Table 4:** Incidence, intensity, and total gap of financial catastrophism due to out-of-pocket payments in health in Spain (EUR). Period 2008–2015.

	“Threshold”	10%	20%	30%	40%
2008	Rate of catastrophic households (H_cat_)	21.32%	10.75%	6.49%	4.40%
	Mean gap of catastrophic households (SD) (O_cat_)	582.04 (2967.03)	410.66 (2756.93)	318.43 (2581.65)	259.84 (2431.55)
	Total gap of catastrophic in Spain (GO_cat_)	9,075,135,678.00	6,357,784,073.00	4,881,702,522.00	3,939,443,519.00
	% Total gap/GDP in Spain	0.82%	0.57%	0.44%	0.36%
2009	Rate of catastrophic households (H_cat_)	19.77%	9.65%	5.76%	4.01%
	Mean gap of catastrophic households (SD) (O_cat_)	529.69 (2680.77)	369.96 (2460.13)	287.98 (2273.78)	235.05 (2111.14)
	Total gap of catastrophic in Spain (GO_cat_)	8,609,916,115.80	6,017,667,479.99	4,697,274,304.20	3,854,970,102.43
	% Total gap/GDP in Spain	0.81%	0.56%	0.44%	0.36%
2010	Rate of catastrophic households (H_cat_)	20.52%	9.95%	5.99%	4.03%
	Mean gap of catastrophic households (SD) (O_cat_)	506.13 (2649.56)	343.80 (2441.01)	259.97 (2271.49)	207.26 (2128.61)
	Total gap of catastrophic in Spain (GO_cat_)	8,481,346,397.97	5,765,895,179.19	4,373,074,098.61	3,497,924,305.76
	% Total gap/GDP in Spain	0.79%	0.54%	0.41%	0.33%
2011	Rate of catastrophic households (H_cat_)	19.54%	9.63%	5.66%	3.78%
	Mean gap of catastrophic households (SD) (O_cat_)	493.06 (2543.39)	335.76 (2329.96)	254.61 (2156.22)	203.13 (2009.25)
	Total gap of catastrophic in Spain (GO_cat_)	8,099,458,828.27	5,468,281,181.50	4,124,061,631.48	3,284,703,463.63
	% Total gap/GDP in Spain	0.76%	0.51%	0.39%	0.31%
2012	Rate of catastrophic households (H_cat_)	19.49%	9.68%	5.85%	3.88%
	Mean gap of catastrophic households (SD) (O_cat_)	476.97 (2323.63)	323.32 (2104.53)	243.09 (1927.98)	192.57 (1779.88)
	Total gap of catastrophic in Spain (GO_cat_)	8,316,818,316.06	5,739,545,398.44	4,393,353,354.19	3,537,520,376.45
	% Total gap/GDP in Spain	0.81%	0.56%	0.43%	0.34%
2013	Rate of catastrophic households (H_cat_)	20.18%	9.73%	5.72%	3.90%
	Mean gap of catastrophic households (SD) (O_cat_)	475.80 (2496.32)	321.20 (2290.36)	242.38 (2124.31)	192.54 (1986.16)
	Total gap of catastrophic in Spain (GO_cat_)	7,811,110,346.00	5,206,533,960.00	3,892,517,415.00	3,063,681,977.00
	% Total gap/GDP in Spain	0.77%	0.51%	0.38%	0.30%
2014	Rate of catastrophic households (H_cat_)	19.85%	9.58%	5.59%	3.85%
	Mean gap of catastrophic households (SD) (O_cat_)	497.79 (2418.05)	338.86 (2197.10)	257.38 (2018.06)	206.84 (1866.71)
	Total gap of catastrophic in Spain (GO_cat_)	9,014,489,924.00	6,251,261,089.00	4,826,132,016.00	3,920,383,390.00
	% Total gap/GDP in Spain	0.87%	0.58%	0.43%	0.34%
2015	Rate of catastrophic households (H_cat_)	20.39%	10.08%	6.11%	4.31%
	Mean gap of catastrophic households (SD) (O_cat_)	543.43 (2626.51)	374.40 (2393.07)	284.01 (2201.01)	226.09 (2038.23)
	Total gap of catastrophic in Spain (GO_cat_)	8,924,322,717.00	6,033,937,813.00	4,502,559,679.00	3,550,531,256.00
	% Total gap/GDP in Spain	0.83%	0.56%	0.42%	0.33%

SD: standard deviation; Hcat is the proportion of households that dedicate more monthly equivalent household income than threshold to out-of-pocket payments in health; Ocat is the average amount dedicated above the threshold of monthly equivalent household income (EUR); GOcat is the global amount dedicated above the threshold of monthly equivalent household income (EUR).

**Table 5:** Marginal effects of the sociodemographic factors of households in Spain associated with financial catastrophism derived from out-of-pocket payments in health in Spain. Year 2008.

	Threshold < 10%			10% ≤ Threshold < 20%			20% ≤ Threshold < 30%			30% ≤ Threshold < 40%			Threshold ≥ 40%		
	dy/dx	SD	*p*-Value	dy/dx	SD	*p*-Value	dy/dx	SD	*p*-Value	dy/dx	SD	*p*-Value	dy/dx	SD	*p*-Value
Female (Ref. male)	−2.58%	0.000	0.000	1.35%	0.000	0.000	0.94%	0.000	0.000	0.12%	0.000	0.000	0.19%	0.000	0.000
Age (Ref. age < 65)															
65–74	−0.15%	0.000	0.000	−0.46%	0.000	0.000	0.24%	0.000	0.000	0.23%	0.000	0.000	0.59%	0.000	0.000
75–84	0.73%	0.000	0.000	−0.07%	0.000	0.000	−0.05%	0.000	0.000	−0.20%	0.000	0.000	0.69%	0.000	0.000
More than 85	−2.23%	0.000	0.000	0.16%	0.000	0.000	−0.13%	0.000	0.000	0.41%	0.000	0.000	1.79%	0.000	0.000
Marital status (Ref. married status)															
Single	5.23%	0.000	0.000	−3.09%	0.000	0.000	−1.26%	0.000	0.000	−0.39%	0.000	0.000	−0.50%	0.000	0.000
Separated/Divorced	0.88%	0.000	0.000	−1.01%	0.000	0.000	−0.48%	0.000	0.000	0.13%	0.000	0.000	−0.48%	0.000	0.000
Widowed	4.83%	0.000	0.000	−2.88%	0.000	0.000	−1.43%	0.000	0.000	−0.03%	0.000	0.000	−0.48%	0.000	0.000
Educational level (Ref. low level (primary school incomplete, primary or equivalent))															
Middle level (secondary school/middle level professional)	−1.29%	0.000	0.000	0.28%	0.000	0.000	0.32%	0.000	0.000	0.47%	0.000	0.000	0.23%	0.000	0.000
University degree or equivalent (university degree or equivalent)	−1.35%	0.000	0.000	0.35%	0.000	0.000	0.20%	0.000	0.000	0.61%	0.000	0.000	0.19%	0.000	0.000
Activity status (Ref. employed)															
Unemployed	−0.66%	0.000	0.000	−0.74%	0.000	0.000	1.51%	0.000	0.000	0.13%	0.000	0.000	0.02%	0.000	0.000
Receiving earnings-related pension	−1.93%	0.000	0.000	0.07%	0.000	0.000	1.14%	0.000	0.000	0.55%	0.000	0.000	0.16%	0.000	0.000
Other situations (homecare, student, etc.)	−2.00%	0.000	0.000	0.08%	0.000	0.000	1.31%	0.000	0.000	0.50%	0.000	0.000	0.06%	0.000	0.000
Household monthly income (Ref. low level income (less than EUR 1200))															
Middle level income (EUR 1200–2500)	4.44%	0.000	0.000	−1.93%	0.000	0.000	−0.89%	0.000	0.000	−0.34%	0.000	0.000	−1.28%	0.000	0.000
High level income (more than EUR 2500)	10.86%	0.000	0.000	−5.94%	0.000	0.000	−1.06%	0.000	0.000	−1.06%	0.000	0.000	−2.81%	0.000	0.000
GDP per capita (Ref. low GDP)															
Middle GDP per capita	−0.72%	0.000	0.000	0.17%	0.00%	0.00%	0.17%	0.00%	0.00%	0.03%	0.000	0.000	0.43%	0.000	0.000
High GDP per capita	−0.76%	0.000	0.000	0.04%	0.00%	0.00%	0.49%	0.00%	0.00%	0.04%	0.000	0.000	0.18%	0.000	0.000
Place of residence urban (Ref. rural)	−0.79%	0.000	0.000	0.17%	0.00%	0.00%	−0.03%	0.00%	0.00%	0.03%	0.000	0.000	0.62%	0.000	0.000
*n*	22,021														
LR χ2 (H0: β1 = β2 = … = βk)	293,898.53														
Prob > χ2	0.000														
Pseudo R2	0.187														
Classification percentage	88.99%														

dy/dx: marginal effect. Includes the slope of the calculated parameter; SD: standard deviation; *p*-value: corresponds to the test of individual significance of the corresponding parameter; LR: corresponds to the test of overall significance of all the slopes in the model; The overall *p*-value was estimated for the complete variables age, marital status, educational level, activity status, household monthly income, and GDP per capita. The *p*-value obtained in all cases was *p*-value = 0.000.

**Table 6:** Marginal effects of the sociodemographic factors of households in Spain associated with financial catastrophism derived from out-of-pocket payments in health in Spain. Year 2011.

	Threshold < 10%			10% ≤ Threshold < 20%			20% ≤ Threshold < 30%			30% ≤ Threshold < 40%			Threshold ≥ 40%		
	dy/dx	SD	*p*-Value	dy/dx	SD	*p*-Value	dy/dx	SD	*p*-Value	dy/dx	SD	*p*-Value	dy/dx	SD	*p*-Value
Female (Ref. male)	−2.38%	0.000	0.000	1.69%	0.000	0.000	−0.12%	0.000	0.000	0.65%	0.000	0.000	0.16%	0.000	0.000
Age (Ref. age < 65)															
65–74	0.64%	0.000	0.000	−0.35%	0.000	0.000	−0.75%	0.000	0.000	0.52%	0.000	0.000	−0.07%	0.000	0.000
75–84	1.83%	0.000	0.000	−1.45%	0.000	0.000	−0.35%	0.000	0.000	0.87%	0.000	0.000	−0.88%	0.000	0.000
More than 85	4.18%	0.000	0.000	−1.61%	0.000	0.000	−2.06%	0.000	0.000	−0.75%	0.000	0.000	0.24%	0.000	0.000
Marital status (Ref. married status)															
Single	13.60%	0.000	0.000	−6.81%	0.000	0.000	−1.99%	0.000	0.000	−1.66%	0.000	0.000	−3.13%	0.000	0.000
Separated/Divorced	7.54%	0.000	0.000	−3.38%	0.000	0.000	−1.47%	0.000	0.000	−0.97%	0.000	0.000	−2.62%	0.000	0.000
Widowed	9.64%	0.000	0.000	−4.67%	0.000	0.000	−1.39%	0.000	0.000	−0.59%	0.000	0.000	−2.09%	0.000	0.000
Educational level (Ref. low level (primary school incomplete, primary or equivalent))															
Middle level (secondary school/middle level professional)	−1.67%	0.000	0.000	1.64%	0.000	0.000	−0.12%	0.000	0.000	0.06%	0.000	0.000	0.10%	0.000	0.000
University degree or equivalent (university degree or equivalent)	−1.29%	0.000	0.000	1.64%	0.000	0.000	−0.32%	0.000	0.000	0.12%	0.000	0.000	0.15%	0.000	0.000
Activity status (Ref. employed)															
Unemployed	−1.70%	0.000	0.000	0.68%	0.000	0.000	−0.33%	0.000	0.000	0.63%	0.000	0.000	0.71%	0.000	0.000
Receiving earnings-related pension	−1.13%	0.000	0.000	−1.06%	0.000	0.000	0.71%	0.000	0.000	−0.11%	0.000	0.000	1.59%	0.000	0.000
Other situations (homecare, student, etc.)	0.66%	0.000	0.000	−1.54%	0.000	0.000	0.69%	0.000	0.000	−1.23%	0.000	0.000	−1.40%	0.000	0.000
Household monthly income (Ref. low level income (less than EUR 1200))															
Middle level income (EUR 1200–2500)	1.54%	0.000	0.000	0.07%	0.000	0.000	−0.83%	0.000	0.000	−0.08%	0.000	0.000	−0.70%	0.000	0.000
High level income (more than EUR 2500)	6.20%	0.000	0.000	−1.30%	0.000	0.000	−2.24%	0.000	0.000	−0.73%	0.000	0.000	−1.93%	0.000	0.000
GDP per capita (Ref. low GDP)															
Middle GDP per capita	1.14%	0.000	0.000	−0.38%	0.00%	0.00%	−0.57%	0.00%	0.00%	0.13%	0.000	0.000	−0.33%	0.000	0.000
High GDP per capita	0.31%	0.000	0.000	−0.31%	0.00%	0.00%	−0.36%	0.00%	0.00%	0.32%	0.000	0.000	−0.67%	0.000	0.000
Place of residence urban (Ref. rural)	0.18%	0.000	0.000	0.80%	0.00%	0.00%	−0.19%	0.00%	0.00%	−0.31%	0.000	0.000	−0.48%	0.000	0.000
*n*	21,625														
LR χ2 (H0: β1 = β2 = … = βk)	355,200.90														
Prob > χ2	0.000														
Pseudo R2	0.143														
Classification percentage	80.45%														

dy/dx: marginal effect. Includes the slope of the calculated parameter; SD: standard deviation; *p*-value: corresponds to the test of individual significance of the corresponding parameter; LR: corresponds to the test of overall significance of all the slopes in the model; The overall *p*-value was estimated for the complete variables age, marital status, educational level, activity status, household monthly income, and GDP per capita. The *p*-value obtained in all cases was *p*-value = 0.000.

**Table 7:** Marginal effects of the sociodemographic factors of households in Spain associated with financial catastrophism derived from out-of-pocket payments in health in Spain. Year 2015.

	Threshold < 10%			10% ≤ Threshold < 20%			20% ≤ Threshold < 30%			30% ≤ Threshold < 40%			Threshold ≥ 40%		
	dy/dx	SD	*p*-Value	dy/dx	SD	*p*-Value	dy/dx	SD	*p*-Value	dy/dx	SD	*p*-Value	dy/dx	SD	*p*-Value
Female (Ref. male)	−0.79%	0.000	0.000	1.31%	0.000	0.000	−0.30%	0.000	0.000	−0.34%	0.000	0.000	0.15%	0.000	0.000
Age (Ref. age < 65)															
65–74	−0.58%	0.000	0.000	−0.10%	0.000	0.000	−0.42%	0.000	0.000	−0.50%	0.000	0.000	0.44%	0.000	0.000
75–84	0.26%	0.000	0.000	0.78%	0.000	0.000	−0.51%	0.000	0.000	−0.73%	0.000	0.000	0.20%	0.000	0.000
More than 85	−0.87%	0.000	0.000	1.33%	0.000	0.000	0.18%	0.000	0.000	−0.96%	0.000	0.000	0.33%	0.000	0.000
Marital status (Ref. married status)															
Single	3.34%	0.000	0.000	−1.27%	0.000	0.000	−1.00%	0.000	0.000	−0.47%	0.000	0.000	−0.59%	0.000	0.000
Separated/Divorced	0.54%	0.000	0.000	1.64%	0.000	0.000	0.51%	0.000	0.000	−0.07%	0.000	0.000	−0.67%	0.000	0.000
Widowed	3.74%	0.000	0.000	−3.26%	0.000	0.000	−0.73%	0.000	0.000	0.41%	0.000	0.000	−0.17%	0.000	0.000
Educational level (Ref. low level (primary school incomplete, primary or equivalent))															
Middle level (secondary school/middle level professional)	−1.22%	0.000	0.000	0.48%	0.000	0.000	0.18%	0.000	0.000	0.12%	0.000	0.000	0.44%	0.000	0.000
University degree or equivalent (university degree or equivalent)	−1.14%	0.000	0.000	0.38%	0.000	0.000	0.89%	0.000	0.000	0.01%	0.000	0.000	0.12%	0.000	0.000
Activity status (Ref. employed)															
Unemployed	0.15%	0.000	0.000	−1.55%	0.000	0.000	1.23%	0.000	0.000	−0.44%	0.000	0.000	0.61%	0.000	0.000
Receiving earnings-related pension	−5.53%	0.000	0.000	1.81%	0.000	0.000	1.32%	0.000	0.000	0.73%	0.000	0.000	1.67%	0.000	0.000
Other situations (homecare, student, etc.)	−5.95%	0.000	0.000	3.74%	0.000	0.000	0.38%	0.000	0.000	0.54%	0.000	0.000	1.29%	0.000	0.000
Household monthly income (Ref. low level income (less than EUR 1200))															
Middle level income (EUR 1200–2500)	3.40%	0.000	0.000	−1.35%	0.000	0.000	−0.81%	0.000	0.000	−0.35%	0.000	0.000	−0.89%	0.000	0.000
High level income (more than EUR 2500)	8.92%	0.000	0.000	−3.75%	0.000	0.000	−2.27%	0.000	0.000	−0.58%	0.000	0.000	−2.31%	0.000	0.000
GDP per capita (Ref. low GDP)															
Middle GDP per capita	−0.04%	0.000	0.000	−0.23%	0.000	0.000	−0.04%	0.000	0.000	0.006%	0.000	0.000	0.37%	0.000	0.000
High GDP per capita	−0.40%	0.000	0.000	0.17%	0.000	0.000	−0.09%	0.000	0.000	0.27%	0.000	0.000	0.59%	0.000	0.000
Place of residence urban (Ref. rural)	−0.72%	0.000	0.000	0.62%	0.000	0.000	0.07%	0.000	0.000	0.02%	0.000	0.000	0.02%	0.000	0.000
*n*	22,054														
LR χ2 (H0: β1 = β2 = … = βk)	285,554.88														
Prob > χ2	0.000														
Pseudo R2	0.162														
Classification percentage	88.52%														

dy/dx: marginal effect. Includes the slope of the calculated parameter; SD: standard deviation; *p*-value: corresponds to the test of individual significance of the corresponding parameter; LR: corresponds to the test of overall significance of all the slopes in the model; The overall *p*-value was estimated for the complete variables age, marital status, educational level, activity status, household monthly income, and GDP per capita. The *p*-value obtained in all cases was *p*-value = 0.000.

## Data Availability

Publicly available datasets were analyzed in this study. This data can be found here: https://www.ine.es/dyngs/INEbase/es/operacion.htm?c=Estadistica_C&cid=1254736176806&menu=resultados&idp=1254735976608#!tabs-1254736194790.

## Supplementary Materials

The following are available online at https://www.mdpi.com/1660-4601/18/3/932/s1, Table S1: Data coding of the explanatory variables.
