# Supplementary material for: Catastrophic Household Expenditure Associated with Out-of-Pocket Healthcare Payments in Spain
Source: Int J Environ Res Public Health. 2021 Jan 21;18(3):932. doi: 10.3390/ijerph18030932 (PMC7908509; doi:10.3390/ijerph18030932)
Supplement: Supplementary file 1 [file ijerph-18-00932-s001.pdf]

Table 1. Data coding of the explanatory variables.

		**Value code**
Gender	Male	0
	Female	1
Age	Age less than 65	0
	65-74	1
	75-84	2
	More than 85	3
Marital Status	Married Status	0
	Single	1
	Separated / Divorced	2
	Widowed	3
Educational level	Low level (Primary school incomplete, primary or equivalent)	0
	Middle level (Secondary school / middle level professional)	1
	University degree or equivalent (University degree or equivalent)	2
Activity Status	Employed)	0
	Unemployed	1
	Receiving earnings-related pension	2
	Other situations (homecare, student, …)	3
Household Monthly Income	Low Level Income (less than €1200))	0
	Middle level income (€1200 - €2500)	1
	High level income (more than €2500)	2
GDP per capita	Low GDP per capita	0
	Middle GDP per capita	1
	High GDP per capita	2
Place of residence	Rural	0
	Urban	1
